# Role of steroids in septic shock: Assessment of knowledge, attitudes and practices among intensivists practising in Hyderabad

**DOI:** 10.4103/0972-5229.58539

**Published:** 2009

**Authors:** Deven Juneja, Palepu B. Gopal, Rashmi R. Satapathy, Ravichandra Raya, Venugopal V. Madgundi

**Affiliations:** **From:** Department of Anesthesia and Critical Care Medicine, Global Hospital, Lakdi-ka-pul, Hyderabad - 04, India

**Keywords:** Knowledge, attitude and practices study, septic shock, steroids

## Abstract

**Context:**

Use of steroids in septic shock is an issue of contention, more so with two major trials reporting conflicting results.

**Aims:**

To assess the current knowledge, attitudes and practices (KAP) related to the role of steroids in septic shock among intensivists practising in Hyderabad.

**Setting, Design, Materials and Methods:**

Questionnaires containing 10 questions pertaining to the role of steroids in septic shock, were distributed to 76 intensivists during the monthly critical care meeting.

**Results:**

A great majority of intensivists (82%) agreed that the role of steroids is restricted to septic shock not responding to vasopressors. There was no clear consensus regarding the role of corticotropin stimulation test or the timing of total cortisol level testing, if it has to be performed. Hydrocortisone was clearly the choice of steroid for most intensivists and intravenous bolus injection being the preferred route of administration. There was no agreement regarding the dose of steroids, the role of fludrocortisone and whether steroids should be tapered. Most of the respondents did not extend the steroid therapy beyond seven days and the most common side effect reported was hyperglycemia.

**Conclusion:**

There is a lot of ambiguity in the knowledge, attitudes or practices regarding role of steroids in septic shock among intensivists in Hyderabad. Uniform policies and protocols need to be devised at institutional level, with multispecialty inputs, and doctors need to be familiarized accordingly.

## Introduction

Despite recent developments in the management of sepsis, the mortality associated with severe sepsis remains high. Cardiopulmonary support in sepsis consists of early use of aggressive fluid resuscitation and, if necessary, use of vasopressors to maintain a mean arterial blood pressure of 65 mm Hg in order to maintain adequate tissue oxygenation. Role of steroids in septic shock has been a contentious issue since 1957, when it was suggested that adrenocortical hormones may be of benefit in shock due to an overwhelming inflammatory response to bacteremia.[1] This issue has come to the forefront again with two recent major trials, the French study by Annane *et al*.[2] and the CORTICUS study,[3] reporting conflicting results. The only thing common to both the trials was that they both showed that use of steroids in septic shock may lead to early withdrawal of vasopressors. The publication of CORTICUS study, almost a year ago, had a worldwide impact on the management of septic shock with major changes in Surviving Sepsis Guidelines[4] as well as international bodies such as Society of Critical Care Medicine (SCCM) and European Society of Intensive Care Medicine (ESICM) coming up with revised guidelines.[5] In keeping with the recent developments, we aimed to assess the current situation about the knowledge, attitude and practices (KAP) related to the role of steroids in septic shock among intensivists practicing in Hyderabad.

## Materials and Methods

The issues pertaining to the role of steroids in septic shock were identified and formulated in a 10-point questionnaire form. Questions covering the following aspects regarding the role of steroids in septic shock were prepared.

- Diagnosis of adrenal insufficiency
- Whom to treat with steroids?
- How to treat?

The questions included

1. What is the role of steroids in sepsis?
  - In all patients with sepsis
  - In all patients with septic shock
  - In septic shock patients not responding to vasopressors
2. Corticotropin (ACTH) stimulation test should be done in
  - All patients with sepsis
  - Few selected
  - None
3. Total cortisol level testing should be done in
  - Morning
  - Evening
  - Random
  - None
4. Which steroids do you use for septic shock?
  - Prednisolone
  - Hydrocortisone
  - Methyl prednisolone
  - Dexamethasone
5. Which is the mode of therapy applied by you?
  - Oral
  - IV bolus
  - IV infusion
6. What is the dose of steroids used by you?
  - 50 mg IV QID hydrocortisone or equivalent
  - 100 mg IV TID hydrocortisone or equivalent
  - 100 mg hydrocortisone followed by 10 mg/h infusion or equivalent
  - Others
7. Oral fludrocortisone (50 μg/day) should be used in
  - All patients
  - Few selected
  - None
8. What is the duration of your steroid therapy?
  - Upto 7 days
  - 7-14 days
  - More than 14 days
  - Till there is clinical improvement
9. Do you taper steroids?
  - Always
  - Never
  - Depending upon duration of treatment
10. What are the common complications experienced by you?
  - GI Bleeding
  - Infection
  - Hyperglycemia
  - Others

The questionnaire was distributed to all the delegates during the monthly meeting of Indian Society of Critical Care Medicine (ISCCM) Hyderabad chapter. The answered questionnaires were collected back at the end of the meeting.

Intensivists were defined as general physicians, pulmonologists or anesthesiologists who were currently involved in taking care of critically ill patients in intensive care units. Postgraduate students in training in the field of intensive care also answered some of the questionnaires.

## Results

A total of 76 intensivists answered the questionnaire, of which 14 (18.4%) were general physicians, 6 (7.9%) were pulmonologists and 56 (73.7%) were anesthetists. The results are given in Table 1.

**Table 1:** Response to various questions regarding the role of steroids in septic shock

Question	Response	Number (%) (n = 76)
What is the role of steroids in sepsis	In all patients with sepsis	1 (1.3)
	In all patients with septic shock	13 (17.1)
	In septic shock not responding to vasopressors	62 (81.6)
Corticotropin stimulation test should be done in	All septic patients	3 (3.9)
	Few selected	42 (55.3)
	None	31 (40.8)
Total cortisol level testing should be done in	Morning	39 (51.3)
	Evening	11 (14.5)
	Random	15 (19.7)
	None	11 (14.5)
Which steroids do you use for septic shock*	Prednisolone	4 (4.8)
	Hydrocortisone	59 (71.1)
	Methyl prednisolone	16 (19.3)
	Dexamethasone	4 (4.8)
Which is the mode of therapy applied by you	Oral	4 (5.3)
	IV bolus	61 (80.3)
	IV infusion	11 (14.5)
What dose of steroids do you use	50 mg iv QID	33 (44.6)
	hydrocortisone or equivalent	
	100 mg iv TID hydrocortisone or equivalent	34 (45.9)
	100 mg hydrocortisone followed by 10mg/hr infusion or equivalent	7 (9.5)
	Others	0 (0)
Oral fludrocortisones should be used in	All patients	1 (1.4)
	Few selected	33 (45.8)
	None	38 (52.8)
What is the duration of your steroid therapy	Up to 7 days	47 (61.8)
	7-14 days	18 (23.7)
	More than 14 days	0 (0)
	Till there is clinical improvement	11 (14.5)
Do you taper steroids	Always	35 (46.1)
	Never	6 (7.9)
	Depending on duration of treatment	35 (46.1)
What are the common complications experienced by you	GI Bleeding	9 (11.8)
	Infection	19 (25)
	Hyperglycemia	46 (60.5)
	Others**	2 (2.6)

* A few intensivists used more than one type of steroids, and hence they were allowed to mark more than one option;

** Others included myopathy and neuropathy reported by one intensivist each

A great majority of intensivists (82%) agreed that the role of steroids is restricted to septic shock not responding to vasopressors. There was no clear consensus regarding the role of adrenocorticotropic hormone (ACTH) stimulation test or the timing of total cortisol level testing, if it has to be performed. Hydrocortisone was clearly the choice of steroid for most intensivists and intravenous bolus injection being the preferred route of administration. There was no agreement regarding the dose of steroids, the role of fludrocortisone or whether steroids should be tapered. Most of the respondents did not extend the steroid therapy beyond seven days and the most common side effect reported was hyperglycemia.

A few intensivists did not answer some questions, but their number was not big enough to change the results. A few intensivists used more than one type of steroids, and hence they were allowed to mark more than one option.

## Discussion

Deficiency of cortisol is associated with increased morbidity and mortality in critical illness. The postulated mechanisms by which steroids may aid patients with septic shock include correction of a state of adrenal insufficiency, inhibition of synthesis of inducible nitric oxide synthase (iNOS) leading to reduced production of nitric oxide and hence lesser vasodilation, restoration of the sensitivity of vascular catecholamine receptors and decrease in transcription of inflammatory cytokines.[6,7] Not only have steroids shown to improve blood pressure but also administration of hydrocortisone during septic shock has been shown to reduce the prevalence of post-traumatic stress disorder and improve the emotional well- being of survivors of septic shock.[8] In spite of these potential advantages, presently steroids are indicated in only those patients with septic shock who have failed to respond to vasopressors.[4]

Adrenal insufficiency, as assessed by the ACTH stimulation test, has been reported in up to 61% of patients with septic shock as compared to 0% in septic patients without shock.[9] Random total cortisol level testing and the ACTH stimulation test are among the commonly employed tests for assessing adrenal insufficiency in critically ill patients. Annane *et al*.[2] recommended that ACTH stimulation test should be done in all patients before starting steroids and they should be discontinued in those patients who test positive to ACTH test. The results from the CORTICUS study[3] indicated that in patients with septic shock, the decision to treat with steroids should be based on clinical criteria and not on the results of adrenal function testing, as the response to steroids cannot be predicted on the basis of this test. Hence, it is recommended that ACTH testing is not necessary for diagnosis of adrenal insufficiency in severely stressed patients, as the central nervous system-hypothalamic-pituitary-adrenal (HPA) axis is already maximally activated in such patients and a random stress cortisol level is a sufficient indicator of the integrity of the entire HPA axis.[5] Although majority of our respondents believed that total cortisol levels should be done in the morning hours, it is not essential to measure the cortisol levels at a specific time of the day as critically ill patients lose their usual diurnal variation in the cortisol levels,[10] and hence random samples can be taken. In spite of the total serum cortisol levels being more representative of the adrenal insufficiency, it is not recommended as it is not widely available and there is lack of data on the levels of cortisol in critically ill patients.[4]

In this study, hydrocortisone was the steroid of choice for 71.1% of respondents, followed by methyl prednisolone, prednisolone and dexamethasone. Although methyl prednisolone, prednisolone and dexamethasone have been used with success in the management of septic shock,[11,12] data regarding their utility is not convincing and the current recommendations are to use hydrocortisone. In addition, use of dexamethasone is not recommended which may be due the fact that it has no mineralocorticoid activity.[13] Methyl prednisolone also has minimal mineralocorticoid activity as compared to prednisolone and hydrocortisone.[13] Annane *et al*.[2] recommended addition of fludrocortisone (50 μg/day) for its mineralocorticoid effect of retaining sodium and water, and hence increasing blood pressure. However, no other study has evaluated its role and the current literature does not support the use of fludrocortisone as hydrocortisone alone has sufficient mineralocorticoid activity. Nevertheless, 46% of respondents in our cohort believed that it might be indicated in a few selected patients.

Steroids may be administered by oral, intravenous bolus or through infusion. More than 80% respondents agreed that they should be administered as bolus injection in divided doses. Oral drugs are generally not advised in patients with severe sepsis on high vasopressor support, as the absorption may be unpredictable. Use of steroid as continuous infusions has been suggested hoping that it may allow better glycemic control. Briegel *et al*.[14] studied the effect of continuous infusion of stress doses of corticosteroids on the duration of vasopressor therapy in patients with septic shock. Although treatment with steroids was associated with a more rapid reversal and a trend towards earlier resolution of organ dysfunction, there were no significant differences in shock reversal or mortality compared to placebo.

Early trials showed that high-dose steroid therapy is detrimental and should not be a component of severe sepsis therapy.[15–17] But interest in steroid use in septic shock was revived when it was shown that moderate doses of steroids started early in the course of septic shock not only reduces vasopressor use but also leads to lesser mortality. Hence, steroids up to 200 mg/day have been recommended as there is no evidence that high dose steroids may have any further benefit and it is also associated with increased adverse effects.[15–17] There was no concordance regarding the dose of steroids in our cohort and 46% were using steroids equivalent to 300 mg hydrocortisone per day.

A majority of our respondents (62%) used steroids up to seven days, but a few (14.5%) agreed that steroids should be continued until there is clinical improvement. There are no clear cut guidelines on the duration of treatment with steroids, but at least seven days of treatment is recommended if the patient does not develop any serious side effects. Although abrupt stopping of steroids after less than 14 days of administration does not cause HPA axis suppression,[18,19] it is recommended that they should be tapered slowly and not stopped abruptly, as abruptly stopping steroids can result in a rebound increase of proinflammatory mediators, with recurrence of the features of shock.[20,21] Furthermore, treatment with steroids causes down regulation of steroid receptors, potentiating the rebound phenomenon with the abrupt cessation of steroid treatment.[22] Only a few intensivists (8%) never tapered steroids, but there was an even splitting of vote between always tapering of steroids and tapering of steroids according to duration of therapy.

Hyperglycemia was reported as the most common side effect by 60.5% of intensivists followed by infection and gastrointestinal bleeding. Steroids can increase the serum glucose levels by increasing the rate of hepatic gluconeogenesis and inhibiting adipose tissue glucose uptake.[23] Other common side effects of steroids include increased rate of infection, gastrointestinal bleeding, fluid retention, myopathy and neuropathy. There is limited literature available on the adverse effect profile of short-term steroid therapy. A meta-analysis found adverse events to be no different from control patients.[24] In the more recent CORTICUS study,[3] patients receiving steroids had an increased incidence of hyperglycemia, superadded infections and bleeding, as compared to patients receiving placebo therapy.

The study attempted to assess the current practices followed by intensivists in regard to the use of steroids in patients with septic shock. The results of this study reflect the uncertainty that exists among the intensivists at various levels regarding the role of steroids in sepsis. Considering the varied and nonuniform practices among intensivists, it seems imperative that just the presence of international guidelines is not enough, but creating awareness among the local intensivists regarding these guidelines is important. Compliance monitoring should be done to check adherence to guidelines, specifically to each component of the sepsis bundle, including the use of steroids. This may require further studies to find out the factors preventing the intensivists from implementing these guidelines and then attempting to modify these international guidelines to suit the local circumstances in a way that they can be used more extensively. Intensivists should be encouraged to formulate policies and protocols at an institutional level, applying an evidence-based approach. Self-monitoring programs and internal audits should be organized to assess the efficacy of the implemented protocols from time to time.

### Limitations

It is obvious that a short 10-point questionnaire cannot cover all issues pertaining to the role of steroids in septic shock. In addition, this study was conducted in a single city, and hence the results may lack wider applicability.

## Conclusion

While enquiring about the KAP level of intensivists regarding the role of steroids in septic shock we found considerable ambiguity. Uniform policies and protocols need to be devised at institutional level, with multispecialty inputs, and doctors need to be familiarized accordingly. Studies on similar context but with wider scope and larger sample size are recommended to confirm findings of this study and to further explore other relevant factors especially factors influencing practice and perception of intensivists regarding role of steroids in septic shock.
